# A Compact Hydraulic Head Auto-Regulating Module (CHARM) for long-term constant gravity-driven flow microfluidics

**DOI:** 10.1038/s41378-025-00968-6

**Published:** 2025-05-29

**Authors:** Fan Xue, Ulri N. Lee, Joel Voldman

**Affiliations:** Massachusetts Institute of Technology, Cambridge, MA USA

**Keywords:** Engineering, Nanoscience and technology

## Abstract

Fluid flow is a ubiquitous aspect of microfluidic systems. Gravity-driven flow is one microfluidic flow initiation and maintenance mechanism that is appealing because it is simple, requires no external power source, and is easy to use. However, the driving forces created by hydraulic head differences gradually decrease during operation, resulting in decreasing flow rates that are undesirable in many microfluidic applications such as perfusion culture, droplet microfluidics, etc. Existing methods to maintain a constant gravity-driven flow either require additional control equipment, involve complex fabrication or operation, are incompatible with miniaturization, or introduce interfaces that lack robustness. Here we tackled those problems by introducing a 3D-printed compact hydraulic head auto-regulating module that automatically maintains a constant fluid level at the microfluidic inlet port without human intervention. Our module successfully maintained a constant hydraulic head for more than 24 h, with the operation time solely limited by the reservoir capacity. A comparison with the conventional gravity-driven flow demonstrated our device’s capability to produce a more stable flow over the perfusion period. Overall, our module creates a simple, robust solution to produce a stable flow rate in gravity-driven flow systems. The compactness of the design allows easy parallelization and compatibility with high-throughput applications, and the biocompatibility of the materials enables the device’s use with life science applications.

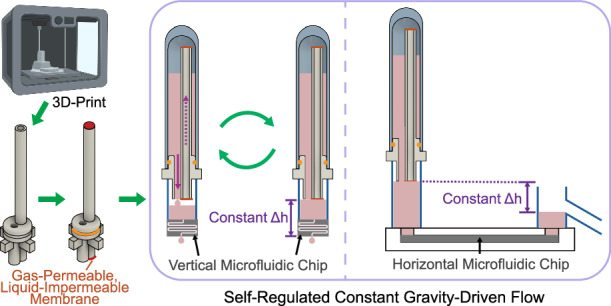

## Introduction

Fluid flow is a ubiquitous aspect of microfluidic systems. Flow mechanisms, depending on the types of driving forces, can generally be categorized into active pumping mechanisms and passive pumpless mechanisms. Active pumping mechanisms for microfluidics involve driving fluid via an external power source/field or actuators^1^. Common active pumps for microfluidics include syringe pumps^2–4^, peristaltic pumps^5,6^, piezoelectric pumps^7–9^, and magnetic pumps^10–12^. While active pumping methods generally provide precise control on the port pressures or flow patterns, they typically require external off-chip controllers, which limits their use for applications where many independent pumps are needed in parallel. Interfacing with external controllers can also introduce bubbles to fluidic channels, and void volumes associated with tubing connections lead to waste of expensive reagents. Some efforts have attempted to mitigate these issues by integrating the pumps onto chips^13–16^, but those pumps still require complex manufacturing procedures and external power sources.

Passive flow techniques, meanwhile, do not require external power sources or actuators. Devices configured for passive flow techniques take advantage of the potential energy of the fluid itself, including osmotic potential energy for osmosis-driven flow^17–19^, surface energy for surface tension-driven flow^20–22^ and capillary-driven flow^23–28^, and gravitational potential energy for gravity-driven flow^29–38^. Those techniques are self-operational without external controls, and are generally more compact and less bubble-prone than the active pumping mechanisms. The elimination of bulky power sources allows easy integration of those flow mechanisms on chip, which reduces the device footprint and decreases the difficulty of fluid handling.

Because of those advantages, passive flow techniques are commonly used in lab-on-a-chip or point-of-care microfluidic systems^1^. That said, they bring their own set of challenges. For example, osmosis-driven flow requires a semi-permeable membrane^1^, and does not have flexibility in fluid concentrations. Surface tension-driven flow and capillary-driven flow depend on the device material and fluid properties, which limits the device’s adaptability to new systems and flexibility to generate self-regulated flows. On the other hand, gravity-driven flow is appealing in its simplicity in both fabrication and operation, because the device needs no extra components, and the driving force can be easily created or modified by altering the relative fluid height between the flow inlet and outlet. Due to its compactness and simplicity, gravity-driven flow is a popular choice for microfluidic designs that need simple flow profiles.

Gravity-driven flow is primarily driven by the hydrostatic pressure difference, $$\begin{array}{c}\varDelta {P}_{{hydrostatic}}=\rho g\varDelta h\end{array}$$, where $$\begin{array}{c}\varDelta h\end{array}$$ is the difference of the fluid height (hydraulic head) between the inlet and outlet, $$\begin{array}{c}\rho \end{array}$$ is the fluid density, and $$\begin{array}{c}g\end{array}$$ is the standard gravity. While the absolute pressure driving the flow also includes the gas pressure difference above the fluid and the surface tension difference generated by curvature of fluid surfaces, those other pressure differences are usually considered negligible, guaranteed by the near-constant atmospheric pressure and the near-flat fluid surfaces at the inlet and outlet reservoirs. Since $$\begin{array}{c}\rho \end{array}$$ and $g$ are usually constants for incompressible fluids like water, people generally alter $\varDelta h$ to alter the driving force.

However, in typical gravity-driven flow, the fluid levels change as time passes by, so the driving pressure varies over time, leading to temporal variations in flow rate, which is a limitation for microfluidic systems that require constant flow. For example, a constant, continuous flow rate is usually needed in cell culture applications where steady media perfusion is needed to maintain a stable micro-environment for cell growth^39,40^, in laminar stream generation applications where the generated streams need to be kept at a specific width^41,42^, and in droplet microfluidics where fluids need to be infused at a constant rate to ensure uniformity of the emulsions^35,43^.

Efforts have been made to maintain a relatively constant flow rate for gravity-driven flow devices, including using extra-wide reservoirs and high-fluidic resistance channels to mitigate the change in fluid heights^38^, utilizing horizontal inlet and outlet reservoirs^32,33^, incorporating a capacitive fluid sensor with feedback control enabled by a microcontroller^34^, placing the chip on a slowly tiling plate that compensates the fluid level change^36^, continuously overfilling the inlet reservoirs and collecting the overflow fluids^35,37^, and using a semi-permeable membrane as a valve to control fluid filling with feedback^31^.

However, these existing methods to maintain constant gravity-driven flow either require additional control equipment^34,36^, need complex fabrication or operation^34^, are incompatible with miniaturization^31,34–38^, increase fluid waste^35,37,38^, or introduce interfaces that lack robustness^31–33^. These features make them not suitable for use with high-throughput, robust applications.

In this work, we introduce a compact hydraulic head auto-regulating module (CHARM) that can be directly placed on top of microfluidic inlet ports as small as the size of a standard 96-well microtiter plate and automatically maintains a constant fluid level at the microfluidic inlet port without human intervention over extended periods. Therefore, when the output port fluid height is also kept constant by draining excessive fluid out from a certain height, the hydraulic head difference, and hence the driving pressure remains the same during the time of operation. The device is purely mechanical and needs no additional power sources, with its operation principle originating from Wang et al.^31^. Without the use of external tubing, our device is less bubble-prone, and more robust than the other tubing-required systems. Fabricated with low-cytotoxicity materials, CHARM can be used with cell culture applications that require constant medium perfusion. Its compatibility with 96-well plates and other compact, multiple-open-access devices enables seamless integration with high-throughput applications. In this paper, we discuss the theory behind operation, experimentally validate the design, demonstrate the device’s capability to generate stable gravity-driven flows, and verify the biocompatibility of the device material.

## Results

### Device structure, principle and theoretical analysis

**Fig. 1:**
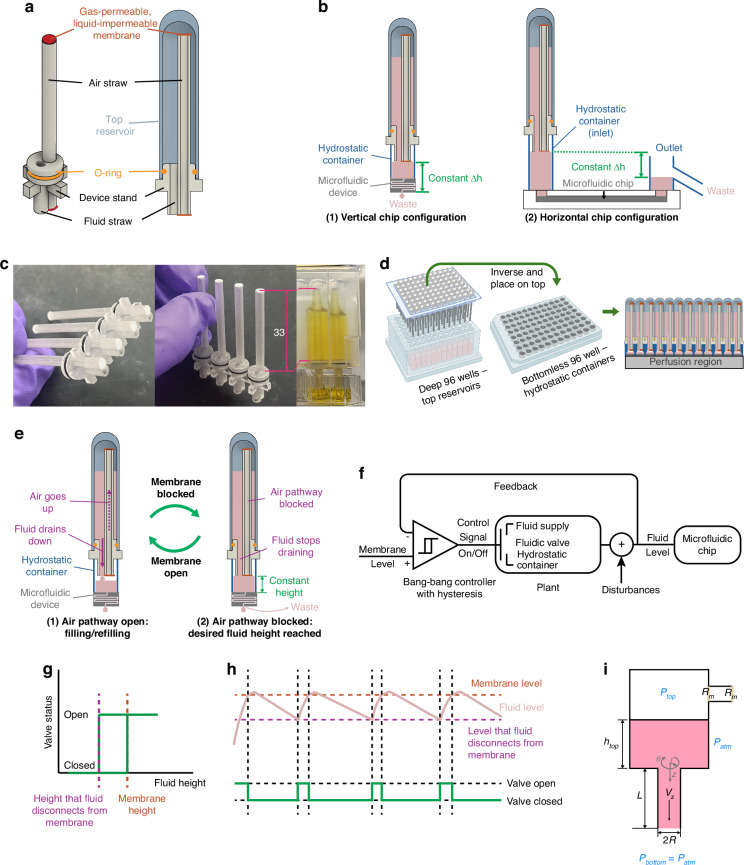
CHARM device structure, configurations, and operation principles. **a** CHARM device structure. Isometric view (left) and cross-sectional view (right). The top reservoir is not shown in the isometric view. **b** CHARM microfluidic chip configurations for a single device showing (1) vertical configuration and (2) horizontal configuration. **c** Fabricated devices. Left and middle: four device bodies (clear) in a row with integrated PTFE membranes (white) and O-rings (black). Right: two devices in operation showing fluid levels reached at the membranes. The dimension is shown in mm. **d** CHARM assembly in 96-well plate format, showing insertion of CHARM system into a deep-well plate that acts as top reservoirs, followed by inversion of the system and placement onto the 96-well plate hydrostatic containers. Created in BioRender. Xue, F. (2025) https://BioRender.com/z05a647 (**e**) CHARM working principle, showing how air goes up into the top reservoir, enabling fluid to drain down, until the fluid level in the hydrostatic container reaches the liquid-impermeable PTFE membrane. Once the membrane is blocked, no more air can enter the top reservoir. This creates a constant-height fluid hydraulic head that can be used to drive a microfluidic device. **f**–**h** CHARM control theory analysis. The system is equivalent to a bang-bang controller with hysteresis that controls the status of a fluidic valve that connects the supply fluid to the hydrostatic container fluid depending on the difference between the fluid level and the membrane level (**f**). The binary-controlled fluidic valve is closed when the fluid level reaches the membrane, and open when the fluid level drops below the point where surface tension can hold up the fluid to the membrane (**g**, **h**). **i** Theoretical model developed for filling/refilling processes

**Fig. 2:**
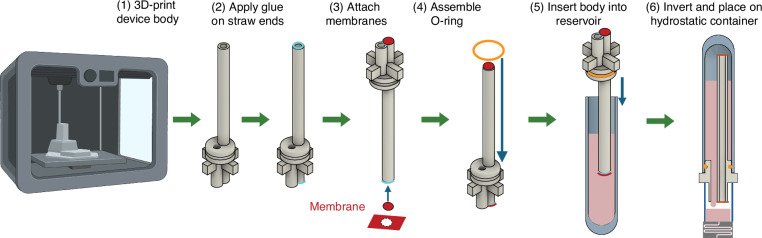
CHARM fabrication method. Created in BioRender. Xue, F. (2025) https://BioRender.com/q82w891

CHARM is composed of a device body with a short fluid straw, a long air straw, and a device stand, two pieces of gas-permeable, liquid-impermeable PTFE membranes attached to both ends of the air straw, a top reservoir, and an O-ring to seal the device body and the top reservoir (Fig. 1a). The device is fabricated by 3D-printing the device body, gluing membranes on the straw ends, and assembling into the top reservoir (Fig. 2). The assembly can be placed on top of a hydrostatic container (microfluidic inlet port) as small as a 96-well microtiter plate to supply fluids and drive the microfluidic chip. The microfluidic device can operate vertically or horizontally (Fig. 1b). Multiple device bodies can be printed in parallel (Fig. 1c), and when using a 96-deep-well microtiter plate as the top reservoirs, the entire assembly can be placed directly on a standard 96-well microtiter plate with microfluidic channels integrated on the bottom (Fig. 1d).

At the initiation of operation, fluid from the top reservoir drains down through the fluid straw to the hydrostatic container, while air goes up through the air straw towards the reservoir to balance the pressure within (Fig. 1e(1)) (modeled in Note S1). The draining continues until the fluid in the hydrostatic container blocks the gas-permeable, liquid-impermeable PTFE membrane, at which point air is prevented from entering the air straw and thus the top reservoir, and, ideally, no more fluid drains (Fig. 1e(2)). In reality, a small amount of overfilling can occur (Note S2, Fig. S1). As fluid flows through the attached microfluidic device, fluid draining from the hydrostatic container will open the air pathway again, allowing more fluid to be supplied from the top reservoir (Fig. 1e(1)). Therefore, through the filling and refilling cycles, a constant fluid height is maintained in the hydrostatic container, set by the membrane level. Notably, the hydrostatic container is always open to the atmosphere as the device stand allows air to pass by freely. This ensures that the gas pressures at the microfluidic inlet and outlet ports are both the atmospheric pressure and do not contribute to the driving pressure for the flow.

In essence, our device is a bang-bang controller that controls a fluidic valve which opens or closes the fluid pathway from the top reservoir to the hydrostatic container (Fig. 1f–h). The controller input is the error signal between the actual fluid level and the membrane level (set point). When the fluid level has not reached the membrane, the controller outputs “on” to the fluidic valve, allowing fluid to fill into the hydrostatic container. The valve is turned off after the fluid reaches the membrane, blocking fluid filling. A slight overshoot can happen (Fig. 1h) because the air in the top reservoir will expand slightly (<1%) after membrane is blocked (Note S2, Fig. S1) and evaporation of the top container fluid can slightly increase the top container gas volume (close to 0) (Note S3, Fig. S2). After the fluid level drops below the membrane, a negative pressure in the air straw prevents the top layer of fluid below the membrane to drop immediately, and surface tension of the fluid can create a curved interface connecting the hydrostatic container fluid to the membrane, until the fluid level drops low enough to break the surface tension, opening the fluidic valve. This delay is modeled as a hysteresis. Due to the binary nature of the controller, the process variable (the fluid level) will oscillate around the set point, though in this case, due to the physical mechanisms in play, the oscillation is not equally around the set point.

Our controller is binary with two states automated by purely passive physical mechanisms. Since no active controls are included, it is not amenable to other types of controllers such as the PID controller, which can provide smoother responses with zero steady-state error. That said, alterations in system design can be used to tune the dynamics, undershoot, and overshoot. For example, because the undershoot happens primarily due to surface tension, one can decrease the undershoot by increasing the fluid temperature and therefore decreasing the surface tension of fluid.

To ensure proper functioning of CHARM, the volumetric filling or refilling rates ($$\begin{array}{c}Q\end{array}$$), i.e., the rate of fluid draining from the top reservoir, should be larger than the microfluidic chip perfusion rate. Modeling the filling/refilling processes as fluid draining from a cylindrical pipe attached to a wide reservoir bottom (Fig. 1i), we derived $$\begin{array}{c}Q\end{array}$$ to be an equation that relates to the device geometry, fluid height and properties, and membrane permeabilities:

1 $$Q=\rho g({h}_{{top}}+L)/\left(\frac{8\mu L}{\pi {R}^{4}}+2* {R}_{m}\right)\,$$

where $$\begin{array}{c}{h}_{{top}}\end{array}$$ is the fluid height in the top reservoir, $$\begin{array}{c}L\end{array}$$ is the fluid straw length, $$\begin{array}{c}\mu \end{array}$$ is the viscosity of the fluid, $$\begin{array}{c}R\end{array}$$ is the fluid straw radius, and $$\begin{array}{c}{R}_{m}\end{array}$$ is the membrane resistance to air flow. The derivation can be found in Note S1. The pressure term, $\rho g({h}_{{top}}+L)$, varies as $$\begin{array}{c}{h}_{{top}}\end{array}$$ decreases, while the resistance term, composed of the straw fluid resistance, $\frac{8\mu L}{\pi {R}^{4}}$, and the membrane resistance, $2* {R}_{m}$, stays constant. ${R}_{m}$ for the particular membranes that we used (POREX Virtek® PMV10 and PMV25) was interpreted from their typical air flow under 70 mbar from the product specifications (107 L/hr/cm² for PMV10 and 17 L/hr/cm² for PMV25), assuming the resistance is independent of the applied pressure. These two membranes were chosen because of their liquid-impermeable characteristics and their reasonably large gas permeabilities, which allows the filling/refilling rate to be much larger than the perfusion rate of most microfluidic systems. The estimated ${R}_{m}$ is 7.52 × 10^9^ N s/m^5^ and 4.72 × 10^10^ N s/m^5^ for PMV10 and PMV25, respectively. This membrane resistance dominates the total resistance term, as the straw fluid resistance in our system is around 3.3 × 10^7^ N s/m^5^, less than 0.5% of the membrane resistance, when water ($$\begin{array}{c}\mu \end{array}$$ ≈ 1 mPa·s at 20 °C) is the fluid to be perfused. Therefore, alterations of fluid viscosity will have negligible effect on the filling/refilling rate unless a fluid with viscosity around ~1000 mPa·s is used (e.g., pure glycerol with $$\begin{array}{c}\mu \end{array}$$ ≈ 1412 mPa·s at 20 °C), in which case the straw fluid resistance increases to ~4.7 × 10^10^ N s/m^5^). The filling/refilling rates over the fluid column height (${h}_{{top}}+L$) calculated using the estimated ${R}_{m}$ assuming viscosities similar to water are 39.24 μL/min/mm and 6.26 μL/min/mm for PMV10 and PMV25 membranes, respectively. Even with very low fluid column heights, those filling/refilling rates are much larger than the flow rates of many microfluidic chips.

To evaluate the effect of fluid evaporation in the closed top reservoir, we calculated the dynamics of volume change caused by evaporated water (Note S3, Fig. S2) from the evaporation equation from Shah^44,45^. Evaporation approaches equilibrium in less than 1 min, and the resulting volume change ( < 30 μL) is negligible.

### Biocompatibility testing

We anticipate that CHARM would be useful for applications where the driven fluid would be in contact with cells or other sensitive components. Thus, we wanted to establish the biocompatibility of the material used in printing the CHARM device. To verify the biocompatibility of the 3D-printing material (MED610^TM^) and justify our devices’ use for life science applications, we performed a 7-day elution test for cytotoxicity on the 3D-printed parts using mouse NIH3T3 fibroblasts and evaluated the material’s effect on cell growth. Four conditions were evaluated: (1) MED610^TM^, (2) polypropylene (PP) as a negative control, (3) no material control (media subjected to the same extraction conditions), and (4) fresh media control.

**Fig. 3:**
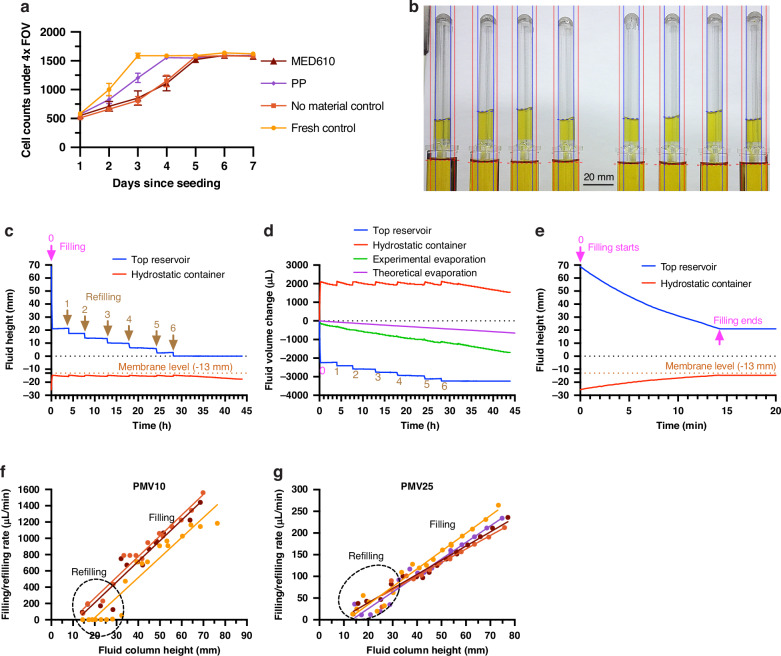
Biocompatibility test cell counts and static operation setup and results. **a** Cell counts under the 4x objective field of view (FOV) during the 7-day cytotoxicity test. Error bars correspond to standard deviations (*N* = 4). **b** Image of 8 CHARM devices being simultaneously operated during static operation. Water level detection regions and detected water levels for top reservoirs (blue) and hydrostatic containers (red). **c** Extracted fluid heights relative to the bottom of the top reservoir measured from one representative device, showing the filling and refilling cycles (0-6) and the constant fluid level in the hydrostatic container (red line). **d** Volume changes converted from the fluid heights in (**c**) and comparing to the theoretical evaporation. The experimental evaporation rate was the sum of the volume changes in both containers. **e** Enlarged view of the filling process in (**c**). **f**, **g** Filling/refilling rates measured from different devices (shown in different colors) and their relationship with fluid column heights, demonstrating the linear relationship between flow rate and pressure head, across devices with high-permeability PMV10 (**f**) and low-permeability PMV25 (**g**) membranes

Cells under all conditions grew from a similar initial count to a similar final count, with some variations in the growth rates (Fig. 3a). Starting from day 5, cells in all conditions reached confluency (Fig. 3a, Fig. S3). The fresh media control condition grew the fastest, the PP condition went next, while the MED610^TM^ and the no material control condition had a similar slowest rate of growth. We ascribe the relatively slower growth rate of MED610^TM^ and no material control to the 5-day incubation of media under 37 °C for material extraction, which could have aged the media. Since MED610^TM^ grew at a similar rate to the no material control extracted under the same conditions, no adverse effects for cell growth were found for the MED610^TM^ material. The results support the validity of using the material for cell culture applications.

### CHARM static operation

An essential feature of CHARM is that it maintains a constant hydraulic head at the inlet of the downstream microfluidic device. We first assessed whether CHARM could maintain a constant hydraulic head in hydrostatic conditions, i.e., when the outlet flowrate approached zero. We set up CHARM devices in hydrostatic containers with water and used time-lapse imaging to measure fluid level over ∼45 h of operation (Fig. 3b). Although the hydrostatic containers were not connected to microfluidic chips, evaporation from the open hydrostatic containers slowly drained fluid from the bottom container, mimicking the effect of an attached low-flowrate microfluidic chip.

The fluid height relative to the bottom of the top reservoir over the 45 h testing time for one representative device and the corresponding volume change are shown in Fig. 3c, d. From the top reservoir fluid level change, an initial filling cycle and several subsequent refilling cycles were observed, indicating that the air pathway was periodically open and blocked, as expected. During each cycle, the water in the top reservoir filled to the hydrostatic container until the hydrostatic container fluid level reached the membrane, and then stayed constant for a period of time until evaporation decreased the hydrostatic container fluid level to open up the membrane. After the fast initial fill (in cycle 0), CHARM kept the fluid height variations within 1.1 mm (corresponding to 10.8 Pa) for > 30 h of operation time until the supply fluid ran out, after which the hydrostatic container fluid level gradually decreased due to evaporation. For better visualization of the processes, the enlarged view during the initial filling process is shown in Fig. 3e. An important finding was that fabrication should ensure that the PTFE membrane does not overhang the air straw, as that provides additional route for air entry, altering device behavior (Fig. S4). Overall, these results demonstrate the ability of the CHARM device to maintain a constant hydraulic head in nearly hydrostatic conditions, with its operation time only limited by the volume of the top reservoir.

To understand whether the decrease in fluid height was plausibly due to evaporation, we used linear regression to extract the experimental evaporation rate of 34 μL/hr (Fig. 3d). The theoretical evaporation rate, calculated using the equation from Shah^44,45^, was ~ 14 μL/hr under the experimental conditions, which was reasonably close to the experimental findings. The remaining discrepancy could be due to the fact that the bottom evaporation surface area was small, in which case the edge effects started to be non-negligible as the humidity around the edge was lower than the humidity it would be if the bottom were also surrounded by fluid. Also, the room air velocity was not exactly zero as used in the theoretical evaporation calculation because of the ventilation system in the room and the lab personnel walking around.

We next sought to evaluate how the semipermeable membrane affected the performance of the device. We determined filling and refilling rates in multiple devices that were fabricated with two different membranes: PMV10 (Fig. 3f) and PMV25 membranes (Fig. 3g), each with a different permeability. The results show a linear relationship with regard to the fluid column height, ${h}_{{top}}+L$, supporting the relationship calculated in Eq.1. In fact, the refilling rates in Fig. 3f were sometimes lower than expected from the linear relationship, especially for the data shown in orange dots. This was probably because there was still some fluid retaining on the membrane when the fluid level dropped in the hydrostatic container, resulting in a higher membrane resistance. The devices with PMV10 membranes had an average slope of 25 μL/min/mm, while those with PMV25 membranes had an average slope of 3.6 μL/min/mm, close to the slopes theoretically interpreted from the membrane specifications (39.2 μL/min/mm for PMV10 and 6.26 μL/min/mm for PMV25). The small discrepancies could be due to several reasons: (1) the membrane resistance, or pressure:flow ratio does not stay the same over different pressures, so the interpreted membrane resistance (under 7000 Pa) does not apply to our experiment (under ~800 Pa); (2) the glue used to assemble the membranes onto the air straw blocked some area of the membrane, resulting in a smaller membrane area, and therefore a higher resistance and a lower filling/refilling rate; and (3) some fluid retaining on the membrane partially blocked the membrane.

### CHARM dynamic operation

**Fig. 4:**
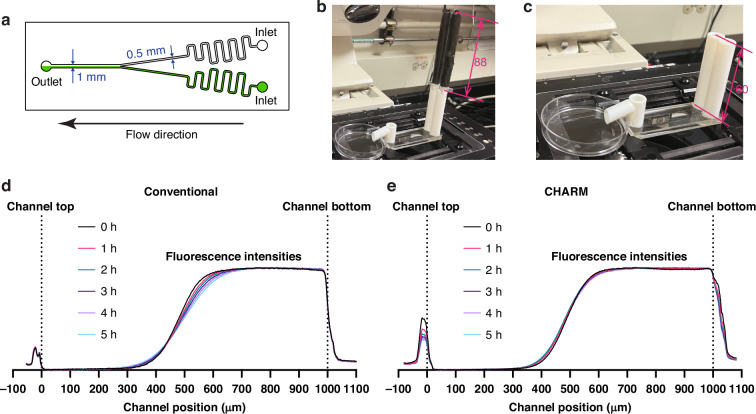
Dynamic operation setup and results. **a** Channel shape used for both conventional gravity-driven flow and CHARM gravity-driven flow experiments. The inlets are on the right, and the outlet is on the left. The bottom channel was perfused with fluorescent fluid (shown in green), while the top channel was non-fluorescent. **b** CHARM gravity-driven flow setup. The CHARM reservoirs were taped to block fluorescent light to dampen photobleaching. The dimension is shown in mm. **c** Conventional gravity-driven flow setup. The dimension is shown in mm. **d**, **e** Fluorescence profile near the channel end during the conventional gravity-driven flow (**d**) and CHARM gravity-driven flow (**e**) operations

Next, we sought to demonstrate the CHARM device’s capability to generate stable gravity-driven flow in a hydrodynamic configuration. To illustrate the importance of constant flow, we setup the CHARM device to feed a Y-shaped microfluidic mixer (Fig. 4a). In such a laminar mixer, the two streams will intermix via diffusion as the two fluids flow down the channel, and thus the slope of the concentration gradient across the stream interface will decrease along the channel. If, however, the flow is not constant, the concentration gradient at a particular location will vary over time. Thus, monitoring the concentration gradient (in this case, via a fluorescence gradient) enables us to assess whether the fluid flow of the two streams is constant.

We used this system to perform a 5 h gravity-driven flow experiment, driven by either the CHARM device (Fig. 4b) or conventional gravity-driven flow (Fig. 4c). We analyzed the laminar stream profile near the outlet of the channel.

For conventional gravity-driven flow, the gradient of the fluorescence intensity at the fluid interface decreases over time (Fig. 4d), indicating more diffusion at the channel end. This is consistent with a decrease in flowrate as the gravity-driven flow hydraulic head decreases. Alternatively, the fluorescence intensities for CHARM gravity-driven flow are more stable (Fig. 4e), indicating the utility of the CHARM device for maintaining constant flow over time.

## Discussion

Our study demonstrates the ability of CHARM to maintain a constant fluid level and produce a stable gravity-driven flow over a prolonged period of time limited solely by the capacity of the reservoir. Compared with other constant flow methods, CHARM does not require external power sources, bulky controllers, or moving mechanical parts. The compactness of our design and its compatibility with the standard 96-well microtiter plate enable parallelized high-throughput experiments. The elimination of external tubing or excessive interfaces reduces chances of failures and increases the robustness of our design. The biocompatibility of the 3D-printing material supported our devices’ use for a variety of life science applications.

The filling and refilling rates were primarily controlled by the membrane air resistance. While a membrane with a lower resistance provides a higher filling/refilling rate, it may provide less water protection. Out of the two membranes used in our experiments, the PMV10 membrane has a higher air permeability than PMV25 membrane, but does not have an ingress protection rating of 68 as does the PMV25 membrane, which suggests that the PMV10 membrane is not guaranteed to protect against permanent submersion in water. If water penetrates the membrane, the air straw can be clogged and the device will stop working. Therefore, there is a trade-off between CHARM filling/refilling rate and the lifetime of the device. A critical premise underlying the device functionality is that the filling/refilling rate should be much larger than the perfusion rate of the microfluidic chip. Therefore, when the perfusion rate allows it, a user can choose a membrane with high water ingress protection—typically with correspondingly larger membrane resistance and thus lower filling/refilling rate—to increase the lifetime of the device.

To avoid cross-contamination between fluids for different experiments, we recommend treating our device as disposable, i.e., replace all components of our device for different experimental runs, including the membrane, device body, O-ring, top reservoir, and hydrostatic container. This disposable characteristic is supported by our device’s low fabrication cost and simple assembly method. However, as no degradation on membranes or other components were observed during our experimental period, one could alternatively clean the device if desired. It should be noted that depending on the specific fluid used, residues can potentially build up and clog the membrane, causing device to be malfunctional. Therefore, membrane permeability needs to be reassessed after cleaning.

Our experiments were performed under room temperature. However, temperature can affect our system through alterations to evaporation dynamics, fluid viscosity, and surface tension. Temperature affects evaporation in two ways. Firstly, a higher temperature increases the evaporation rate from the hydrostatic container, as quantified in the evaporation equation from Shah^44,45^. This effect is equivalent to having a microfluidic chip with a higher perfusion rate, and does not influence CHARM’s ability to maintain a constant hydraulic head as long as the filling/refilling rate remains reasonably higher than the total rate of fluid draining from the hydrostatic container. If this evaporation from the hydrostatic container is unwanted, it can be minimized by having a smaller (close to 0) surface area of fluid opening to the air. Secondly, a higher air temperature increases the saturation water pressure, and consequently increases the theoretical maximum evaporation volume in the top reservoir (Note S3). However, as described in Note S3, the total volume increase in the top reservoir caused by evaporation is close to 0, so little overfill will occur because of this effect even when the temperature increases to typical incubator temperatures (e.g., 37 °C). Temperature also affects viscosity, and various mathematical models and empirical measurements exist for the correlation between fluid viscosity and temperature^46^; from room temperature to 37 °C, water viscosity will decrease by < 2×. As discussed with the device operation principle in the Results section, viscosity change on this order will have negligible effect on system performance. Finally, temperature also affects surface tension. For typical aqueous liquids, the coefficient of surface tension decreases with increasing temperature^47^. Surface tension can affect the timing when fluid breaks apart from the membrane and opens up the air pathway as the fluid level drops in the hydrostatic container. While determination of the exact mechanism of how surface tension affects the fluid behavior near the membrane is out of the range of this study, it is expected that as the temperature increases and surface tension decreases, the fluid will be more readily released from the membrane, so the total variations on the fluid height (i.e., the magnitude of hysteresis for CHARM control) will be smaller.

We noted that the membrane integration method could impact the device behavior, and another water level pattern usually appeared due to air entry from the side of the membrane when the membrane was not cut to the right size or shape and had overhanging regions around the fluid straw (Fig. S4). In fact, even with the side-entry issue, the fluid level stayed relatively constant after the initial water filling. The total fluctuation after the initial filling and before the top container fluid ran out was ~1.54 mm, corresponding to 15 Pa. Therefore, for systems that do not need accuracies below 15 Pa, the overhanging membrane may not be a problem. This gives users of our device more freedom in fabrication when their systems are not sensitive to small fluctuations.

An important feature of our device is compactness, which enables high-throughput experiments. However, this feature also creates a limitation for our system, because the reservoir capacity, and therefore operation time without manual addition of fluids can be limited. For users seeking to achieve a longer operation time or a higher flow rate to the microfluidic chip, the top reservoir and fluid straw dimensions may need to be modified to hold a larger amount of fluid and supply fluid at a higher rate. Another limitation lies in the smallest amount of fluid the device can operate with. While the theoretical largest capacity or flow rate are unlimited, a too low fluid height will greatly reduce the filling/refilling rate of CHARM, which may not be able to sustain the flow rate of the microfluidic chip. Small volumes also increase the effect of surface tension, which can alter the device behavior. While the smallest volume for our system is undetermined, users need to be cautious about using fluid volumes smaller than around 500 μL.

So far, we have only used water in our systems, but for fluids with particular solvents or non-aqueous solutions, it would be important to test the interactions of the fluids with the PTFE membrane to see if (1) the solution penetrates the membrane under the necessary operating conditions, or (2) adsorption of important molecules occur. Surface tension between the fluid and the membrane could also be investigated. A too-low contact angle could result in fluid sticking to the membrane and blocking the air pathway even when the fluid level dropped below the membrane level. Fortunately, PTFE is a material known for its good chemical resistance and non-wetting properties, so many fluids should be compatible in our system.

While CHARM offers a simple solution for constant-flow gravity-driven microfluidics, its small hydraulic head fluctuations limit its applicability in systems requiring ultra-precise flow rates. Our experimental results showed fluctuations within 1.1 mm, corresponding to a 10.8 Pa variation in hydrostatic pressure when using water, setting the resolution of our system. For applications sensitive to fluctuations below this threshold, CHARM is less suitable than other gravity-driven systems with tighter tolerances, such as controllers with electrical sensors that can provide submillimeter fluid height resolutions^34^. Another limitation of our system lies in the fact that CHARM does not provide active flow rate controls, so no temporal flow patterns can be generated. When time-varying flows are needed, other controllers that have active flow rate control are better choices.

Overall, our CHARM system provided a simple, easy-to-fabricate, robust, compact, and high-throughput method to maintain the fluid level at gravity-driven flow microfluidic ports. The applications of the system include but not limited to constant media perfusion for cell/organoid culture, uniform generation of droplets, and chemical synthesis processes. In the future, more applications can be demonstrated, and large-scale fabrication methods can be developed for our system.

## Materials and methods

### CHARM device fabrication

As shown in Fig. 2, the device body was printed using PolyJet Technology with Stratasys J5 Medijet® printer using Stratasys MED610^TM^ transparent rigid biocompatible dental resin. POREX Virtek® PTFE membranes (PMV10 or PMV 25) were punched into 3 mm diameter discs and attached to the ends of the air straw using a thin layer of super glue (Loctite Super Glue Gel Control), and the sides of the membranes were sealed with Bondic UV epoxy glue. An O-ring (The O-Ring Store N1.00×005) was secured on the device body, which was then inserted into a top reservoir container filled with the fluid to be perfused. The top reservoir was either directly cut out from a 96-deep well plate (Thomas Scientific 23A00L817) or 3D printed into a deep well shape with an 8.4 mm inner diameter opening (similar to the opening of the 96-deep well plate) using the same technology as printing the device body. In the case of the 3D-printed top reservoir, the O-ring was replaced by UV glue that fully sealed between the top reservoir and the device body because the 3D-printed top reservoirs have rougher surfaces than the 96-deep well plate, and air might leak through the O-ring. To have a larger fluid supply, the experiments described in this paper all used the 3D-printed reservoir with a larger height than the 96-deep well plate. However, we also fabricated devices with 96-deep wells as top containers (Fig. 1c). The device assembly was inverted and placed on top of the hydrostatic container to start supplying fluid.

### CHARM static operation

CHARMs were placed on top of 15.5 cm ID, 18 cm OD glass tube hydrostatic containers. A total of *N* = 3 devices with PMV10 membranes and *N* = 4 devices with PMV25 membranes were tested. Water used in the experiment was room-temperature and was mixed with yellow food color for better visualization. The hydrostatic container was filled with water to a level below the membrane but not too far away from the membrane before starting the experiment. Water was injected into the top reservoir from the fluid straw using a syringe. After placing the devices in their correct orientations on top of the hydrostatic container, the devices were allowed to sit for ~2 days. The whole filling and refilling processes were videoed using a time-lapse camera with one image taken every 10 s, until the devices stopped filling down fluids. The air temperature and relative humidity level around the device was videoed together and later used in the evaporation calculations.

To analyze the videos taken, fluid levels in both the top reservoirs and the hydrostatic containers were extracted from each frame by detecting color changes. The fluid level changes were converted to volume changes by multiplying the cross-sectional areas, and the evaporation volume was calculated by adding the volume changes in both containers.

To calculate the filling rates, the initial filling processes were divided into 10 regions having the same durations. For each region, a linear fit was performed on the volume change graph to calculate the filling rate, and the fluid column height, defined as the sum of the top reservoir fluid height (${h}_{{top}}$) and the fluid straw height ($L$ = 13 mm), was recorded at the middle of the region. During each refilling cycle, since the refilling duration and fluid height changes were small, one linear fit was performed, and the fluid column height at the middle point was recorded. The filling/refilling rates were plotted against the fluid column heights, and a linear fit was performed on each device.

### CHARM dynamic operation

The microfluidic chip was fabricated by stacking a bottom glass substrate layer, a middle double-sided adhesive channel layer (100 µm height), and a top acrylic port layer. The channel layer and port layer were laser-cut into the desired shape. The port and substrate layers were plasma-treated before bonding. The inlet and outlet ports were 3D-printed using PolyJet Technology with Stratasys J5 Medijet® printer using DraftWhite™ and attached to the top port layer using Bondic UV epoxy glue. The inlet ports had a height of 60 mm while the outlet port had a height of 10 mm from the bottom to the lowest point of the side opening.

Following chip fabrication, the channel was first perfused with 70% ethanol and DPBS for cleaning. At the start of the experiment, the top inlet port was filled with DPBS, while the bottom inlet port was filled with 5 μg/mL FITC fluorescein (Invitrogen™ F1907) in DPBS. For conventional gravity-driven flow, both inlet ports were fully filled, while for CHARM gravity-driven flow, both inlets were filled to ~10 mm below the highest level and CHARM devices with the same fluids as the inlets were placed on top to fill the inlet fluid levels to the membranes. The outlet ports were filled with DPBS until overflow occurred through the side openings. A small petri dish was used to collect the waste coming out of the outlet ports.

Fluorescent images near the channel end were taken every 5 min for 5 h under a 4x objective. The fluorescence profiles across the channels were calculated from the images by averaging the longitudinal fluorescence intensities in the field of view.

### Biocompatibility testing

An elution test for cytotoxicity was performed where the target material (MED610^TM^) was extracted in cell culture media which was later used to grow cells (NIH3T3 fibroblasts passage # 9). The media used was Dulbecco’s Modified Eagle Medium (Gibco, cat#11960044) supplemented with 10% FBS (Gibco cat#16140071), 2% L-glutamine (Gibco cat#25030081), and 1% penicillin-streptomycin (Gibco cat#15140122).

Four conditions were evaluated as stated in the Results section above. The MED610^TM^ used in the experiments were in the shapes of 4 device bodies, while the polypropylene was used in 4 tubing shapes that were cut to the length of the device bodies plus some extra small pieces to make the total surface area roughly equal to the MED610^TM^’s surface area.

The following cleaning procedures were done to the materials before evaluation: (1) sonicate in 70% ethanol for 30 min, (2) soak in new 70% ethanol at room temperature for 6 days, (3) rinse with sterile deionized (DI) water once, (4) sonicate in new sterile DI water for 30 min, (5) rinse with new sterile DI water for 3 times and (6) soak in media in 37 °C incubator for 5 days.

After the cleaning procedures, the MED610^TM^ and polypropylene materials were sub- merged in 40 mL new media, separately, and placed in the 37 °C incubator for 7 days together with another 40 mL of no material control media. All media was then aliquoted into 7 day’s use as cell culture replacement media and stored in a 4 °C fridge before use (3 days before the use of day 1 media). The fresh media was also aliquoted into the same amount as the other 3 conditions.

3T3 cells were seeded into 16 wells (4 independent cell cultures for each of the 4 conditions) in the middle of a 48 well plate in 300 μL fresh media with an initial density of 7500 cells/well. The region surrounding the 16 wells were filled with 300 μL of Dulbecco’s Phosphate-Buffered Saline (DPBS) to create similar humidity environments for the cell culture wells.

On each day starting from the next day after seeding, phase images were taken using Nikon Eclipse Ti-E microscope under both 4x and 10x objectives, and media in each well was replaced with new warmed conditioned media. Cell counts were generated from the 4x images by the software iCLOTS^48^.

## Supplementary information

Supplementary Information
