# Supplementary material for: A Compact Hydraulic Head Auto-Regulating Module (CHARM) for long-term constant gravity-driven flow microfluidics
Source: Microsyst Nanoeng. 2025 May 29;11:113. doi: 10.1038/s41378-025-00968-6 (PMC12122956; doi:10.1038/s41378-025-00968-6)
Supplement: Supplementary file 1 — Supplementary Information [file 41378_2025_968_MOESM1_ESM.docx]

**Note S1: Derivation for CHARM Filling/Refilling Rates**

When the device was initially filling into the hydrostatic container, the system can be considered as fluid draining out from a wide reservoir (top reservoir) through a cylinder attached vertically to the bottom of the reservoir (Fig. 1f). The gas in the top reservoir is connected to the atmosphere by a channel with membranes of resistance ($\begin{aligned} R_{m} \end{aligned}$) attached on both ends.

Assuming the fluid is continuous and incompressible, the following two equations (Eq.S1,S2)^1^ apply to the fluid in the cylinder:

1. Continuity Equation in cylindrical coordinates:

|  | $\begin{aligned} \frac{1}{r}\frac{\partial}{\partial r}(rv_{r})+\frac{1}{r}\frac{\partial}{\partial\theta}(v_{\theta})+\frac{\partial}{\partial z}(v_{z})=0 \end{aligned}$ | (S1) |
| --- | --- | --- |

where $v_{r}$, $v_{\theta}$, and $v_{z}$ are the fluid velocities in the radial, circumferential, and longitudinal directions, respectively, and $r$ is the distance of the point of interest to the central axis of the cylinder.

1. Navier-Stokes equation for the longitudinal (z) component in cylindrical coordinates:

|  | $\begin{aligned} \begin{aligned} \rho(\frac{\partial v_{z}}{\partial t}+v_{r}\frac{\partial v_{z}}{\partial r}+\frac{v_{\theta}}{r}\frac{\partial v_{z}}{\partial\theta}+v_{z}\frac{\partial v_{z}}{\partial z})=-\frac{\partial P}{\partial z}+\rho g_{z}+\mu(\frac{1}{r}\frac{\partial}{\partial r}(r\frac{\partial v_{z}}{\partial r})+\frac{1}{r^{2}}\frac{\partial^{2}v_{z}}{\partial\theta^{2}}+\frac{\partial^{2}v_{z}}{\partial z^{2}}) \end{aligned} \end{aligned}$ | (S2) |
| --- | --- | --- |

where $\rho$ is the fluid density, $g_{z}$ is the gravitational acceleration in the z direction, and

$\mu$ is the fluid viscosity.

Assuming steady flow ($\begin{aligned} \partial v_{z}/\partial t=0 \end{aligned}$), no flow in the radial or circumferential directions ($\begin{aligned} v_{r}=0 \end{aligned}$ and $\begin{aligned} v_{\theta}=0 \end{aligned}$), and axisymmetric flow ($\begin{aligned} \partial v_{z}/\partial v_{\theta}=0 \end{aligned}$), Eq. 2.1 becomes $\begin{aligned} \text{d}v_{z}/\text{d}z=0 \end{aligned}$,

which implies that the flow is fully developed, and Eq. S2 can then be simplified to

|  | $\begin{aligned} \begin{aligned} \frac{\partial P}{\partial z}=\mu\frac{1}{r}\frac{\partial}{\partial r}(r\frac{\partial v_{z}}{\partial r})+\rho g_{z} \end{aligned} \end{aligned}$ | (S3) |
| --- | --- | --- |

Assuming uniform pressure drop across the cylinder of length L, the pressure gradient can be expressed as

|  | $\begin{aligned} \begin{aligned} \frac{\partial P}{\partial z}=-\frac{\Delta P}{L} \end{aligned} \end{aligned}$ | (S4) |
| --- | --- | --- |

where $\begin{aligned} \Delta P \end{aligned}$, expressed in the following equation, is the pressure difference between the top end and bottom end of the cylinder without considering the gravity of the fluid in the cylinder

|  | $\begin{aligned} \Delta P=\rho gh_{top}+P_{top}-P_{bottom} \end{aligned}$ | (S5) |
| --- | --- | --- |

and $h_{top}$ is the height of the top reservoir fluid level measured from the bottom of the top reservoir.

It can be inferred from device configuration that $\begin{aligned} P_{bottom}=P_{atm} \end{aligned}$, and assuming the membrane has negligible resistance to air and $P_{top}$ is instantaneously balanced to $P_{atm}$ when the membrane is not blocked, the above equation becomes

|  | $\begin{aligned} \Delta P=\rho gh_{top} \end{aligned}$ | (S6) |
| --- | --- | --- |

Therefore, Eq. S4 becomes

|  | $\begin{aligned} \frac{\partial P}{\partial z}=-\rho g\frac{h_{top}}{L} \end{aligned}$ | (S7) |
| --- | --- | --- |

Assume the cylinder is perfectly vertical, then $g_{z}$ in Eq. S3 is equal to the standard gravity, $g$ (= 9.81 N/kg). Plugging this relation and Eq. S7 into Eq. S3 gives

|  | $\begin{aligned} -\rho g\frac{h_{top}}{L}=\mu\frac{1}{r}\frac{\partial}{\partial r}(r\frac{\partial v_{z}}{\partial r})+\rho g \end{aligned}$ | (S8) |
| --- | --- | --- |

Two boundary conditions below apply for the system:
(1) Finite flow in the center of the cylinder: $v_{z}$ is finite at $\begin{aligned} r=0 \end{aligned}$, and
(2) No slip at wall: $\begin{aligned} v_{z}=0 \end{aligned}$ at $\begin{aligned} r=R \end{aligned}$, where $R$ is the radius of the cylinder.

Integrating Eq. S8 with the boundary conditions above results in the following flow profile:

|  | $\begin{aligned} v_{z}(r)=(\frac{\rho g(h_{top}/L+1)}{4\mu})(R^{2}-r^{2}) \end{aligned}$ | (S9) |
| --- | --- | --- |

The volumetric flow rate down the cylinder ($Q$) can then be calculated as the integration

of the above equation over the cross-sectional area of the cylinder:

|  | $\begin{aligned} Q=\frac{\pi R^{4}}{8\mu L}\rho g(h_{top}+L) \end{aligned}$ | (S10) |
| --- | --- | --- |

This equation is analogous to the Hagen-Poiseuille equation^2^:

|  | $\begin{aligned} Q=\frac{\pi R^{4}}{8\mu L}\Delta P \end{aligned}$ | (S11) |
| --- | --- | --- |

where the pressure term, $\Delta P$, is replaced by the pressure difference between the fluid level in the top reservoir and the bottom end of the cylinder, $\rho g(h_{top}+L)$. In fact, the discussed derivation process is similar to that for the Hagen-Poiseuille equation, except that an extra gravity term, $g_{z}$, remains in the Navier-Stokes equation. Lemons et al.^3^ reached the same conclusion in a similar system as well, although the derivation process was a little different.

If air cannot pass through the membrane freely ($\begin{aligned} R_{m}>0 \end{aligned}$), then a pressure drop will build up across the membrane, causing the pressure above the fluid level in the top reservoir to decrease, resulting in a lower filling speed.

Assume the pressure drop across the membrane ($\begin{aligned} \Delta P_{air} \end{aligned}$) changes linearly with the air flow across the membrane ($\begin{aligned} Q_{air} \end{aligned}$), then $\begin{aligned} R_{m}=\Delta P_{air}/Q_{air} \end{aligned}$ is a constant and can be added to Eq. S10 as an extra resistance component:

|  | $\begin{aligned} Q=\rho g(h_{top}+L)/(\frac{8\mu L}{\pi R^{4}}+2*R_{m}) \end{aligned}$ | (S12) |
| --- | --- | --- |

with the factor of 2 indicating the presence of 2 membranes. This resistance can be measured for the specific membrane used.

**Note S2: Volume Changes after Membrane Blockage**

After the membrane is blocked, $R_{m}$ goes to infinity, and the drop of pressure in the top reservoir ($P_{top}$) can no longer be balanced by the atmospheric pressure ($P_{atm}$), so $P_{top}$ decreases until the pressure driving the filling reaches zero. The equilibrium happens when

|  | $\begin{aligned} (P_{top}+\rho g(h_{top}+L))-(P_{atm}+\rho gh_{bottom})=0 \end{aligned}$ | (S13) |
| --- | --- | --- |

where $h_{bottom}$ is the overfilling height in the hydrostatic container (Fig. S1). At this point, $Q$ in Eq. S12 reaches zero with $\begin{aligned} R_{m}\to\infty\end{aligned}$.


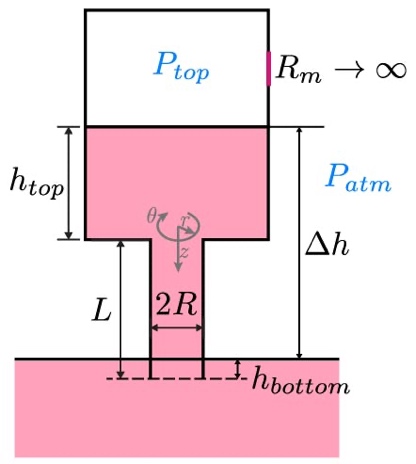


**Fig. S1**. Theoretical model used when the membrane on the straw bottom is blocked. The overfilling height ($\begin{aligned} h_{bottom} \end{aligned}$) was exaggerated for better visualization.

Let $\begin{aligned} \Delta h \end{aligned}$ be the relative height difference between the fluid level from the top and hydrostatic containers, then

|  | $\begin{aligned} \begin{aligned} \Delta h=h_{top}+L-h_{bottom} \end{aligned} \end{aligned}$ | (S14) |
| --- | --- | --- |

So Eq. S13 becomes

|  | $\begin{aligned} P_{top}=P_{atm}-\rho g\Delta h \end{aligned}$ | (S15) |
| --- | --- | --- |

The ideal gas law states that

|  | $\begin{aligned} PV=nR_{const}T \end{aligned}$ | (S16) |
| --- | --- | --- |

where $P$, $V$, and $T$ are the gas pressure, gas volume, and gas temperature, respectively. $R_{const}$ (= 8.314J/mol · K) is the ideal gas constant.

Let $\begin{aligned} V_{orig} \end{aligned}$ be the original gas volume in the top reservoir and the air straw combined, and $\begin{aligned} V_{new} \end{aligned}$ be the new gas volume at equilibrium. Since the amount of gas ($\begin{aligned} n_{gas} \end{aligned}$) stays constant in the top reservoir and air straw after the bottom membrane is blocked, and we assume no change in $T$,

|  | $\begin{aligned} P_{atm}V_{orig}=P_{top}V_{new}=n_{gas}R_{const}T=\text{constant} \end{aligned}$ | (S17) |
| --- | --- | --- |

assuming $\begin{aligned} P_{top}=P_{atm} \end{aligned}$ before the membrane was blocked.

The following relationship between $V_{orig}$ and $\begin{aligned} V_{new} \end{aligned}$ can be derived from Eq. S15 and

S17:

|  | $\begin{aligned} V_{new}=\frac{P_{atm}}{P_{atm}-\rho g\Delta h}V_{orig} \end{aligned}$ | (S18) |
| --- | --- | --- |

Therefore,

|  | $\begin{aligned} \Delta V=V_{new}-V_{orig}=\frac{\rho g\Delta h}{P_{atm}-\rho g\Delta h}V_{orig} \end{aligned}$ | (S19) |
| --- | --- | --- |

Depending on the device height used and the current working condition, $\Delta h$ varies, but is within 5 cm for the proposed system. Therefore, substituting ∆h with $\begin{aligned} \Delta h_{max}=0.05 \end{aligned}$ m, and plugging in $P_{atm}$ = 101325 Pa, $g$ = 9.81 N/kg, and $\rho$ = 1000 kg/m3 to Eq. S19 gives

|  | $\begin{aligned} \Delta V_{max}=\frac{490.5}{101325-490.5}V_{orig}=0.00486V_{orig} \end{aligned}$ | (S20) |
| --- | --- | --- |

This indicates that the gas volume increase in the top reservoir and the air straw is less than 1%. If the hydrostatic container and the top reservoir has the same diameter, the overfill height in the hydrostatic container will be less than 1% of the height of the air column in the top reservoir, which is equal to $\begin{aligned} V_{orig}/A_{top} \end{aligned}$. This results in less than 1 mm change in the overfill height. In practice, the membrane resistances dominate the resistance term during filling/refilling, so the pressure drop across the membrane was high, and $\begin{aligned} P_{top} \end{aligned}$ was smaller than $\begin{aligned} P_{atm} \end{aligned}$ before the membrane was blocked. This resulted in an even less volume change. Therefore, the overfilling height after membrane blockage was negligible.

**Note S3: Theoretical Evaporation in the Closed Top Reservoir**

When the air pathway is blocked and the decrease of pressure in the top reservoir holds up the fluid from further filling down to the hydrostatic container, we want to make sure that the water vapor evaporated to the top reservoir is not going to increase the gas volume too much to allow excessive overfilling in the hydrostatic container.

Assume that evaporation starts after the equilibrium in Eq. S15 is reached. Evaporation of water in the top reservoir is going to increase the amount of water vapor, $\begin{aligned} n_{w} \end{aligned}$, in the top reservoir until the net evaporation rate reaches zero (relative humidity reaches 100%). On the other hand, the amount of dry air in the top reservoir, $\begin{aligned} n_{air} \end{aligned}$, is going to stay the same. The pressure in the top reservoir needs to stay roughly the same as both the old pressure and the new pressure need to satisfy Eq. S15. Although $\Delta h$ in the equation will be smaller due to evaporation, the change in $\rho g\Delta h$ (∼10 Pa/mm) is much smaller than $P_{atm}$. Therefore, in the following calculations, $P_{top}$ is assumed to be a constant.

Applying the ideal gas law (Eq. S16) to water vapor and dry air separately, and assuming the relative humidity to be $\begin{aligned} rh \end{aligned}$ before considering evaporation, the following equation applies to the conditions before considering evaporation (Eq. 2.21) and after evaporation has reached the steady state (Eq. 2.22):

|  | $\begin{aligned} n_{w\_orig}=\frac{rh*P_{ws}}{P_{top}-rh*P_{ws}}n_{air} \end{aligned}$ | (S21) |
| --- | --- | --- |

|  | $\begin{aligned} n_{w\_new}=\frac{100\%*P_{ws}}{P_{top}-100\%*P_{ws}}n_{air} \end{aligned}$ | (S22) |
| --- | --- | --- |

where $P_{ws}$ is the saturation water pressure.

Since $n_{air}$ stays the same, the change in the amount of gas can be expressed as

|  | $\begin{aligned} \Delta n_{total}=\Delta n_{w}=(n_{w\_new}-n_{w\_orig})=\frac{P_{top}}{P_{top}-P_{ws}}\frac{1-rh}{rh}n_{w\_orig} \end{aligned}$ | (S23) |
| --- | --- | --- |

Applying the ideal gas law to both $\begin{aligned} n_{total} \end{aligned}$ and $\begin{aligned} n_{w\_orig} \end{aligned}$ in the above equation gives the total volume change in the top reservoir:

|  | $\begin{aligned} \Delta V_{total}=(1-rh)\frac{P_{ws}}{P_{top}-P_{ws}}V_{orig} \end{aligned}$ | (S24) |
| --- | --- | --- |

$P_{ws}$ can be calculated using the Buck Equation^4^ below:

|  | $\begin{aligned} P_{ws}=611.21*\exp(18.678-\frac{T_{C}}{234.5})(\frac{T_{C}}{257.14+T_{C}}) \end{aligned}$ | (S25) |
| --- | --- | --- |

where $T_{C}$ is the air temperature in °C.

Approximating $P_{top}$ as 101 kPa,

|  | $\begin{aligned} \Delta V_{total}=k(1-rh)V_{orig} \end{aligned}$ | (S26) |
| --- | --- | --- |

where the coefficient $k=\frac{P_{ws}}{101 kPa -P_{ws}}$ ranges from 0.00609 to 0.06630 for top reservoir air temperature from 0°C to 37°C, and $k$ = 0.03238 when $T_{C}$ = 25°C.

This is a less than 6.7% change of the volume of gas in the top reservoir even if the temperature is high (37°C) and the humidity is 0%. In reality, the top reservoir is under a very humid environment after it is filled with supply fluid, and after the initial filling cycle, the subsequent air that passes through the membrane and get into the top reservoir is near saturation (rh ≈ 100%) because the bottom membrane is close to the water level in the hydrostatic container. Therefore, in following refilling processes, $\begin{aligned} \Delta V_{total} \end{aligned}$ is close to 0 and little overfill will occur because of evaporation.

In order to know the dynamics of the evaporation process, the following equation can be solved:

|  | $\begin{aligned} \frac{\text{d}n_{w}}{\text{d}t}=\frac{A_{top}}{M_{w}}E(n_{w},t) \end{aligned}$ | (S27) |
| --- | --- | --- |

where $\begin{aligned} A_{top} \end{aligned}$ in our system is 47.12 mm^2^ determined by the top reservoir cross-sectional area minus the straw end area, $\begin{aligned} M_{w} \end{aligned}$ is the molar mass of water, and the evaporation rate per unit area $\begin{aligned} E(n_{w},t) \end{aligned}$, expressed in kg/(m2·h), is given by

|  | $\begin{aligned} E(n_{w},t)=35AD_{ws}(AD_{r}(P_{w}(n_{w}))-AD_{ws})^{1/3}(SH_{ws}-SH_{r}(P_{w}n_{w})) \end{aligned}$ | (S28) |
| --- | --- | --- |

according to the evaporation equation from Shah^5,6^, where $AD$ is the air density in kg/m^3^, $SH$ is the specific humidity, and the subscripts r indicates room condition and ws indicates saturated condition at the water surface.

Assuming $\begin{aligned} P_{top}=101 \end{aligned}$ kPa, and 25°C water and air temperature, $\begin{aligned} P_{ws} \end{aligned}$ can be calculated from Eq. S25. Then $\begin{aligned} AD \end{aligned}$ and $\begin{aligned} SH \end{aligned}$ in the above equation can be calculated from pressures according to the following two equations, respectively,

|  | $\begin{aligned} AD=0.028964*\frac{P_{total}-P_{w}}{R_{const}T_{air}} \end{aligned}$ | (S29) |
| --- | --- | --- |

|  | $\begin{aligned} SH=0.622*\frac{{P_{w}}/{P_{total}}}{1-\frac{P_{w}}{P_{total}}+0.622*\frac{P_{w}}{P_{total}}}*100\% \end{aligned}$ | (S30) |
| --- | --- | --- |

then $P_{w}$ can be expressed as:

|  | $\begin{aligned} P_{w}=\frac{n_{w}}{n_{w}+n_{air}}P_{top} \end{aligned}$ | (S31) |
| --- | --- | --- |

Solving Eq. S27 numerically where the initial $n_{w}$ value is calculated with the assumption that $V_{orig}$ = 0.8 mL, and converting the result into gas volume gives the solutions as in Fig. S2. The total volume change at steady state ($\begin{aligned} t\to\infty\end{aligned}$) in the solution is verified to be the same in Eq. S26.

It can be seen from the figure that evaporation reaches steady state within ∼1 min, and the total volume change is smaller than 30 μL.


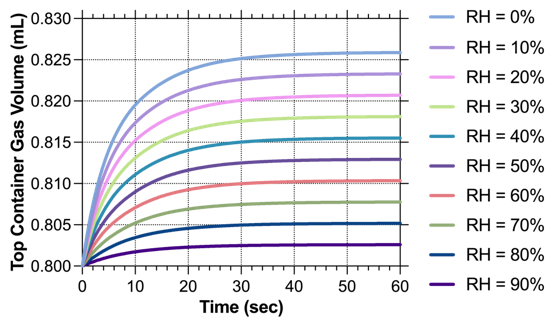


**Fig. S2**. Evaporation dynamics in the top reservoir across different humidity levels assuming an initial gas volume of 0.8 mL, an equal water and air temperature of 25 °C, and a constant total gas pressure of 101 kPa.

**a**
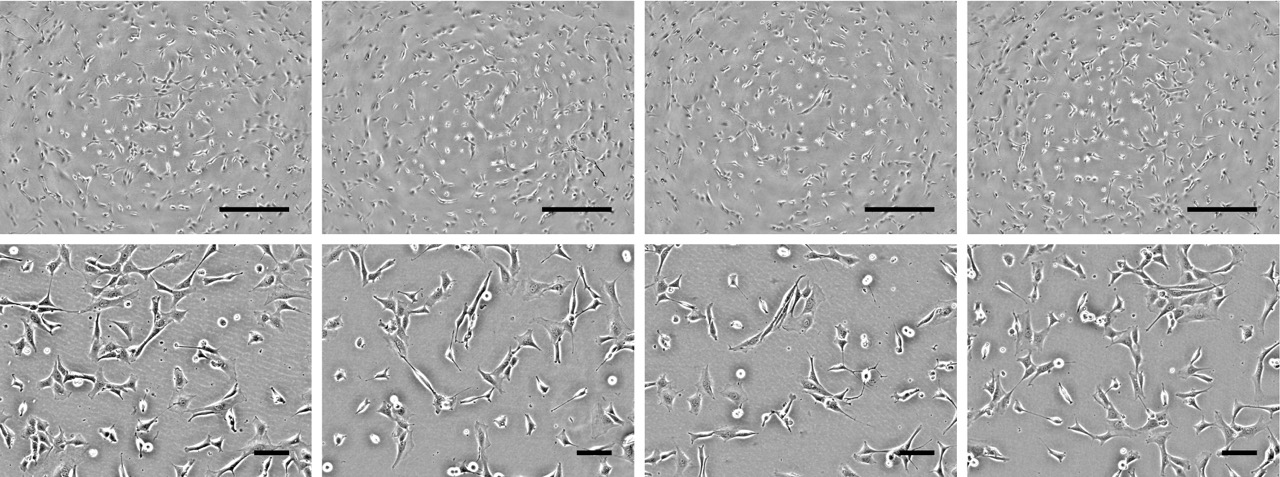


**b**
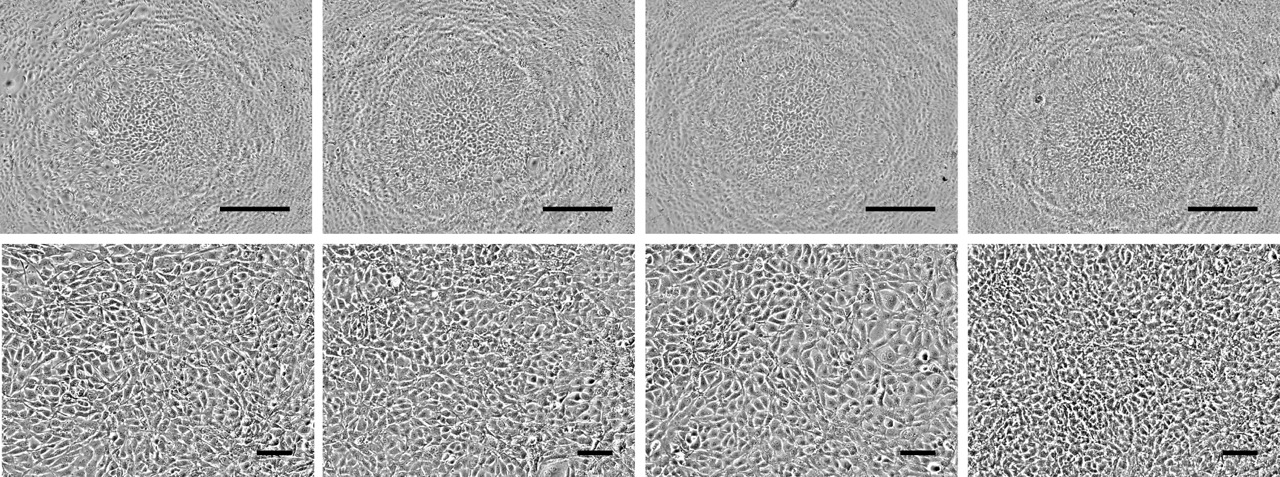


**Fig. S3**. Representative phase images from the cytotoxicity test. For each row of image from left to right, the images are from the MED610^TM^, PP, no material control, and fresh control conditions. The 4 images in each column are from the same well. All images were processed with contrast and brightness adjustments and background removal (by subtracting low-frequencies from the images). (a) Day 1 images under 4x (top row, scale bar = 500 μm) and 10x (bottom row, scale bar = 100 μm). (b) Day 5 images under 4x (top row, scale bar = 500 μm) and 10x (bottom row, scale bar = 100 μm).


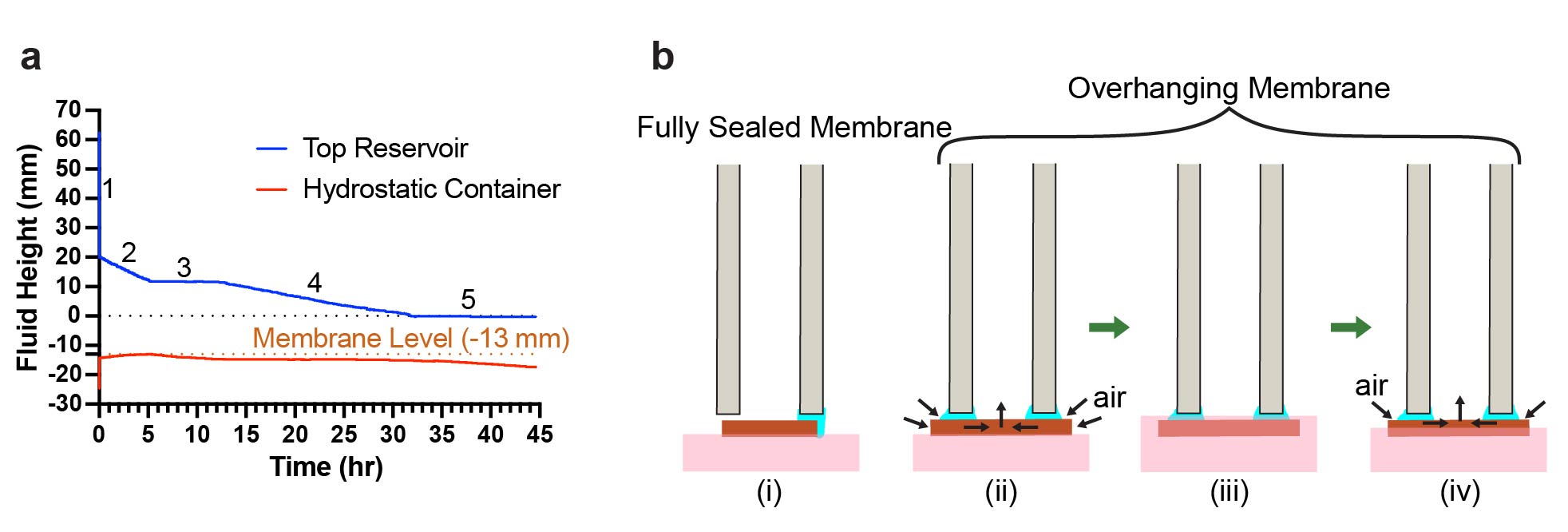


**Fig. S4**. Overhanging membrane regions around the straw end altered device behavior. (a) Fluid pattern of one device with over-hanging membranes showing five stages during operation: (1) fast filling, (2) slow overfilling, (3) no filling, (4) slower continuous filling, and (5) top container fluid ran out. Initially, the fluid in the top reservoir filled down similarly (stage 1). However, the blockage of the membrane bottom did not fully stop the fluid filling, and a slower rate of overfilling was observed, indicating that some air was slowly getting into the top reservoir (stage 2). After the overfilling reached certain height, the overfilling stopped, and a no-fill stage (stage 3) started, which indicated that the air pathway was now blocked. After the bottom fluid level dropped down, a continuous refilling stage (stage 4) began, indicating that air was slowly but continuously filling into the top reservoir. In this stage, the bottom water height stayed roughly constant, indicating that the refilling rate roughly equaled to the evaporation rate. In the end, the top reservoir fluid ran out and the refilling stopped (stage 5). (b) Membrane side-entry hypothesis illustration explaining air pathways to enter the straw. The glue is shown in cyan, membrane in red, straw walls in grey, and water in pink. The fully sealed membrane did not allow air to enter once the bottom was blocked (i). However, the overhanging membrane regions created additional pathways that allow air to enter the straw. When the fluid blocked the bottom entry of air after stage 1, air could still enter the top reservoir slowly from the side, causing a slow overfilling (ii, stage 2). When the overfilling made the bottom fluid level high enough to block the sideway entries, no air could enter the top reservoir and the no-fill stage began (iii, stage 3). Afterwards, when evaporation and microfluidic chip perfusion lowered the fluid level and exposed some membrane area, refilling gradually occurred until the refilling rate approximately equal to the evaporation rate (iv, stage 4). In the end, the top reservoir fluid ran out and the refilling stopped (stage 5).

**References**

1. Roselli, R. J. & Diller, K. R. General Microscopic Approach for Biofluid Transport. in *Biotransport: Principles and Applications* (eds. Roselli, R. J. & Diller, K. R.) 389–485 (Springer, New York, NY, 2011). doi:10.1007/978-1-4419-8119-6_7.

2. Roselli, R. J. & Diller, K. R. Macroscopic Approach for Biofluid Transport. in *Biotransport: Principles and Applications* (eds. Roselli, R. J. & Diller, K. R.) 169–317 (Springer, New York, NY, 2011). doi:10.1007/978-1-4419-8119-6_5.

3. Lemons, D. S., Lipsombe, T. C. & Faehl, R. J. Vertical quasistatic Poiseuille flow: Theory and experiment. *Am. J. Phys.* **90**, 59–63 (2022).

4. Buck, A. L. New Equations for Computing Vapor Pressure and Enhancement Factor. *J. Appl. Meteorol. 1962-1982* **20**, 1527–1532 (1981).

5. Shah, M. M. Analytical formulas for calculating water evaporation from pools. *ASHRAE Trans.* **114**, 610–619 (2008).

6. Shah, M. M. Improved method for calculating evaporation from indoor water pools. *Energy Build.* **49**, 306–309 (2012).
